# Segmental arterial mediolysis: a rare cause of an acute abdomen

**DOI:** 10.1093/jscr/rjab370

**Published:** 2021-10-16

**Authors:** Czara A Kennedy, Desmond P Toomey

**Affiliations:** Department of General and Colorectal Surgery, Midland Regional Hospital Mullingar, Mullingar, Co. Westmeath, Ireland; Department of General and Colorectal Surgery, Midland Regional Hospital Mullingar, Mullingar, Co. Westmeath, Ireland; Department of General and Colorectal Surgery, Mater Misericordiae University Hospital, Dublin, Ireland

## Abstract

Segmental arterial mediolysis (SAM) is a nonatherosclerotic, noninflammatory and nonimmune arteriopathy of unknown aetiology. We present the case of a 43-year-old male who presented to the emergency department with abdominal pain. A computed tomography of abdomen and pelvis showed a narrow, hypodense superior mesenteric artery after the origin, raising the possibility of thrombus or vasculitis. He was commenced on rivaroxaban and steroids. He subsequently presented with an acute abdomen in a collapsed state. Repeat imaging of his abdomen and pelvis revealed an ischaemic ileal segment and caecum. He required an emergency laparotomy with resection of the ischaemic segment and formation of a double-barrelled stoma. SAM is an important diagnosis for clinicians and radiologists to be aware of, given the risks of life-threatening haemorrhage and acute organ ischaemia. This is a commonly overlooked cause of abdominal pain, where an early diagnosis with lifestyle modifications may prevent disease progression and subsequent development of life-threatening complications.

## INTRODUCTION

Segmental arterial mediolysis (SAM) was first described in the literature by Slavin in 1976 [1]. Originally named as segmental mediolytic arteritis, the disease was renamed following observation that the pathological process was different from that of arteritis [2]. SAM is a nonatherosclerotic, noninflammatory and nonimmune arteriopathy of unknown aetiology [3]. It results in dissection, aneurysm, occlusion or stenosis of medium- to large-sized vessels due to lysis of the arterial media [4]. It tends to affect the medium-sized splanchnic and renal arteries. The disease commonly affects patients in the fifth or sixth decades of life, with a slight predisposition for males. SAM was initially thought to be a rare entity; however, case reports are increasing likely due to the widespread use of computed tomography (CT) and CT angiography [5]. SAM is an important diagnosis for clinicians and radiologists to be aware of, given the risks of life-threatening haemorrhage and acute organ ischaemia.

## CASE REPORT

A 43-year-old male presented to the emergency department with sudden onset epigastric pain, radiating to the umbilical region. The pain was associated with nausea and vomiting. At presentation, the patient was afebrile and hypertensive at 160/103 mmHg. On initial examination, the patient’s abdomen was soft with mild tenderness in the epigastric region. Bowel sounds were normal, and no masses or organomegaly were palpable. He had a 13-pack year history of smoking. An urgent CT abdomen and pelvis was ordered.

**Figure 1:**
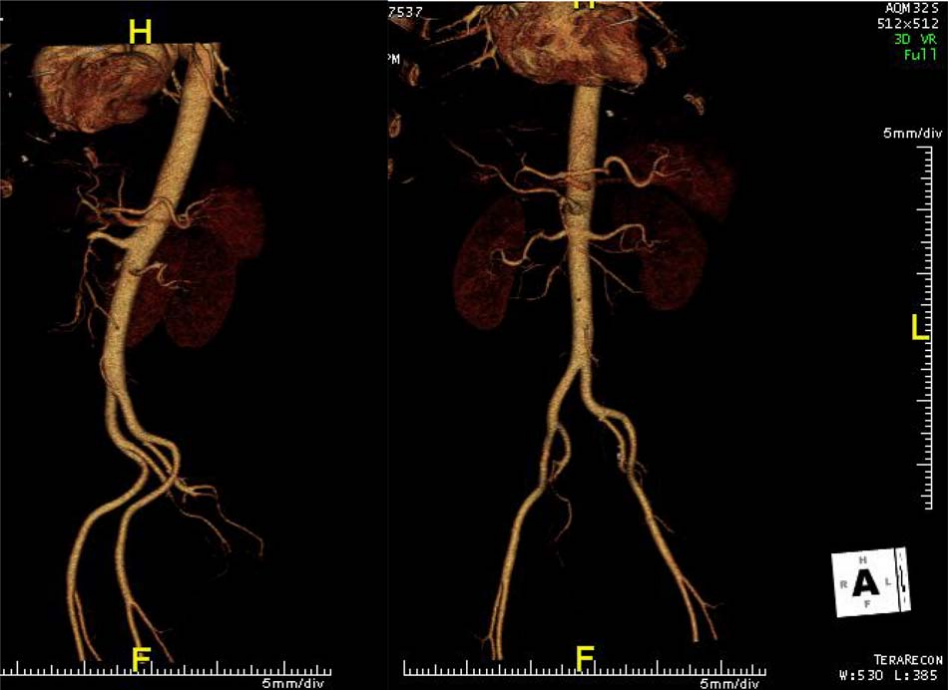
CT angiogram of abdominal aorta (sagittal plane on the left and coronal plane without bones on the right) demonstrating a narrow, hypodense SMA after the origin, raising the possibility of thrombus.

The initial blood count and metabolic panel are shown in Table 1. A CT abdomen and pelvis demonstrated a narrow, hypodense superior mesenteric artery (SMA) after the origin, raising the possibility of thrombus. A CT angiography was subsequently performed, which demonstrated fat stranding around the SMA with nonatherosclerotic narrowing of the artery. There was post-stenotic dilatation and a dissection flap evident (Fig. 1).

**Table 1:** Blood count and metabolic panel at presentation

Laboratory tests	Results
Haemoglobin	14.8 g/dl
White cell count	14.9 × 10^9/l
Neutrophils	11.46 × 10^9/l
Platelets	314 × 10^9/l
Urea	8.24 mmol/l
Creatinine	76 μmol/l
Sodium	140 mmol/l
Bilirubin	8.8 μmol/l
ALP	74 U/l
ALT	33 U/l
GGT	39 U/l
Troponins	<0.01 μg/l
C-reactive protein	2.9 mg/l
Erythrocyte sedimentation rate	9 mm/h
Albumin	41.4 g/l
Amylase	43 U/l
Lactate	2.5 mmol/l
Total cholesterol	6.96 mmol/l

Our initial working diagnosis was vasculitis based on the findings from the CT angiography. Other differential diagnoses included mesenteric angina or a mesenteric thromboembolic event. Following discussion with the consultant rheumatologist and vascular teams, the patient was commenced on therapeutic low molecular weight heparin (LMWH) and intravenous (IV) steroids. A vasculitic screen was sent which was negative (Table 2). The patient was discharged on a dose of rivoroxaban, 20 mg once/day, and a tapering dose of oral steroids.

**Table 2:** Vasculitic and thrombosis screen

Laboratory tests	Results (range)
Antinuclear antibodies	Negative
Smooth muscle antibodies	<1 IU/ml
Mitochondrial antibodies	Negative
Parietal cell antibodies	Negative
Neutrophil cyto antibodies	Negative
Antiphospholipid antibodies	
B2-glycoprotein	1.50 U/ml (0.00–6.99)
Cardiolipin antibodies	Negative
HIV, hepatitis B and C	Not detected
JAK2 mutation	Negative
Complement levels	Normal
Flow paroxysmal nocturnal haemoglobinurea panel	Negative

He represented within 1 week of discharge with severe abdominal pain, which was post-prandial in nature. During this visit, a repeat blood count, metabolic panel and lactic acid levels were all within the normal ranges. A repeat CT abdomen and pelvis was unchanged from his initial imaging. He was placed on bowel rest, commenced on total parental nutrition (TPN), IV unfractionated heparin and IV steroids and was accepted for transfer to the vascular and rheumatology specialities. At this time, our working diagnosis was mesenteric angina secondary to an SMA thrombosis/vasculitis.

His imaging was discussed at the regional multi-disciplinary team meeting, where a diagnosis of SAM was made based on imaging findings and exclusion of other causes. The vascular team recommended medical management as his anatomy was not amenable to bypass surgery or endovascular repair. The patient was discharged on a tapering dose of steroids and LMWH with outpatient follow-up.

The following month the patient presented to a Model 4 hospital with an acute abdomen in a collapsed state secondary to septic shock. A repeat CT of his abdomen and pelvis, including CT angiogram, showed an ischaemic ileal segment and caecum. He was brought to theatre for a laparotomy, ileo-caecal resection and creation of a double-barrelled stoma (jejunum and ascending colon). Two-hundred centimetres of small bowel remained *in situ* from the duodenum-jejunal flexure. The histology showed ischaemic necrosis suggestive of a low flow state with no evidence of thrombosis or vasculitis, confirming the diagnosis of SAM. His stoma was subsequently reversed 20 months post-emergency laparotomy, restoring intestinal continuity. Despite the anatomical concerns regarding the mesenteric blood flow, the patient encountered no post-operative complications.

## DISCUSSION

We present the case of a 43-year-old male who presented to our ED with non-specific abdominal pain. This case highlights the difficulty in diagnosing SAM, and the importance of clinician awareness. The most common acute presentation is abdominal pain, as SAM frequently involves the splanchnic vessels but carotid and retroperitoneal arteries have also been reported [6]. Skeik *et al.*, reported that the most common abdominal artery associated with SAM was the SMA (53%), followed by the hepatic artery (45%), celiac artery (36%), renal artery (26%) and the splenic artery (25%) [5]. Clinical presentation ranges from abdominal pain suggestive of mesenteric angina to acute intra-abdominal haemorrhage or organ ischaemia [7].

Histological diagnosis remains the gold standard to confirm the diagnosis of SAM. However, with the advancement in CT angiography and magnetic resonance angiography, more cases are being diagnosed based on a combination of imaging and clinical criteria [5]. This has reduced the need for an arterial biopsy, which is a high-risk and challenging procedure [8]. The most common finding on CT angiogram is dissection of the mesenteric vessels. Other findings include nonatherosclerotic arterial mural wall thickening with a multifocal skip pattern of luminal strictures and post-stenotic aneurysmal dilatation of arteries. Imaging findings can be similar to inflammatory vasculitis, and therefore autoimmune work-up should be performed to rule out such causes [9].

The pathology of SAM consists of two phases: the initial insult, followed by the remodelling and restoration phase. The initial phase begins with mediolysis of the arterial media. Subsequently, a tear develops, which separates the outer media from the adventitia layer. This process can encompass a portion, or the entire circumference of an artery, and it has a segmental distribution which leaves normal arterial segments in between [3, 10]. After the initial mediolysis, the remodelling and restorative phase starts with granulation tissue, which is subsequently replaced by fibrotic tissue, which ultimately restores its original shape [11]. There is no inflammatory component which distinguishes it from vasculitis.

No standardized guidelines exist at present for the management of patients with SAM due to the lack of randomized controlled trials. Lifestyle modification, control of hypertension and dyslipidaemia should be commenced to reduce the cardiovascular risk profile. Endovascular interventions are recommended as first-line treatment modality in patients with haemodynamic instability or evidence of end-organ ischaemia [12].

## CONFLICT OF INTEREST STATEMENT

The author(s) declared no potential conflicts of interest with respect to the research, authorship, and/or publication of this article.

## FUNDING

This research did not receive any specific grant from funding agencies in the public, commercial or not-for-profit sectors.

## COMPLIANCE WITH ETHICAL STANDARDS

All ethical standards were complied with while conducting this research.

## INFORMED CONSENT

Informed consent was obtained for this paper. The manuscript is not under consideration elsewhere. The work is original. All authors have agreed to this submission, and all authors have seen, edited and approved the final version of this paper.
